# Health Tract, No. 224

**Published:** 1865

**Authors:** 


### WORTH KNOWING.

In household economy a great mistake is often made in the oversight of the fact that the same number, or measure, or weight of the same article, does not always give the same amount of yield, or nutriment.

In every three tons of coal, stove, range, or grate, passing your door from different yards, and to the casual observer all looking exactly alike, there is a difference in their heat-producing value up to as high as one half. Some coals clinker badly, others contain a great many thin flat pieces, but when put in the fire turn white; coal dealers call this "bone," as it has something of the color of burnt bone, it has no coal in it, and is a clear loss. Good coal will not have three pieces of "bone" in a whole day's burning; sometimes the grate is half full of these white pieces by bedtime.

Eggs are of different sizes. In any basket of eggs, the twelve largest and smallest will make a difference of perhaps one third or more.

When we purchase apples by the bushel, we get about the same number of pounds whether they be large or small, and so with potatoes, but there is more nourishment in the Mercer than in the Cusco variety, yet it is to the interest of the farmer to cultivate the "Cusco," even if he sold them at half price, because planting each variety in the same soil, one acre will yield ninety-one bushels of the Jersey Mercer, while two hundred and forty bushels of the Cusco potato was the product of the adjoining acre, tilled in the same manner, as reported by Mr. Williams, at the Farmers' Club of the American Institute, in New-York City.

A piece of "roast beef," in the process of cooking, loses fifteen per cent; if boiled, it loses only eleven per cent. If a leg of mutton is roasted, it loses twenty-five per cent, but only ten per cent, if boiled. So that if you want a "roast" for dinner, beef is cheaper than mutton at the same price per pound, although mutton is four per cent more nutritious than beef.

*Wood.*—Very few persons are aware of the wide difference between the amount of heat yielded by the different qualities of wood, and as wood is sold by measurement, while its value for giving out warmth is determined by its weight, each kind being equally seasoned and dry, it is well to be posted as to these points.

One cord of dry hickory wood will keep up a certain amount of heat for one hundred days, while a cord of pitch pine will last only thirty-five days, and a cord of white oak ninety-one days; a ton of Lehigh coal will last ninety-one days. "Charcoal is charcoal," all kinds are alike as to color; but a ton of pine charcoal lasts seventy-five days; maple, one hundred and fourteen days; oak, one hundred and sixty-six. In the light of these statements, families may save a good many dollars every year.

